# *Myo*-Inositol, *Scyllo*-Inositol, and Other Minor Carbohydrates as Authenticity Markers for the Control of Italian Bulk, Concentrate, and Rectified Grape Must

**DOI:** 10.3390/molecules28083609

**Published:** 2023-04-20

**Authors:** Mauro Paolini, Matteo Perini, Letizia Allari, Loris Tonidandel, Fabio Finato, Katia Guardini, Roberto Larcher

**Affiliations:** 1 Fondazione Edmund Mach (FEM), Via E. Mach 1, 38098 San Michele all’Adige, Italy; 2 Unione Italiana Vini Servizi (UIV), Viale del Lavoro 8, 37135 Verona, Italy

**Keywords:** characterization, *myo*-inositol, *scyllo*-inositol, minor carbohydrates, grape must, data bank

## Abstract

*Myo*-inositol polyalcohol is a characteristic component of natural and concentrated grape musts (CMs), and Regulation (EU) no. 1308/2013 prescribes its presence as a marker of the authenticity of rectified concentrated must (RCM). Other polyalcohols besides *myo*-inositol, such as *scyllo*-inositol or minor sugars, could be considered authenticity markers, but an extensive search in the literature yielded no exhaustively investigated study of their concentration variability in genuine products. The aim of this study was to create an extensive national data bank of minor carbohydrates profiles and investigate the impact of the geographical origin and the different vintages on the concentration of these compounds; to this end, 450 authentic Italian grape musts of different varieties were sampled and analyzed during the harvest season in 2019, 2020, and 2021. The grape musts from the Italian wine-growing areas CII and CIIIb had *myo*- and *scyllo*-inositol contents always higher than 756 and 39 mg/kg of sugar, respectively. Conversely, also considering other mono- and disaccharides, sucrose, sorbitol, lactose, maltose, and isomaltose showed contents always lower than 534, 1207, 390, 2222, and 1639 mg/kg of sugar, respectively. The general applicability to the CM and RCM of the proposed authenticity thresholds, established in the must, was demonstrated by studying the influence of must concentration on the *myo*- and *scyllo*-inositol content. Inter-laboratory comparison experiments were also conducted to harmonize and characterize laboratory methods and validate the analytical dataset. Based on the obtained results, the text of the EU legislation (Reg. (EU) 1308/2013), which defines the characteristics of the must and the CRM product, should be revised.

## 1. Introduction

Enrichment is an oenological practice designed to increase the natural alcoholic strength of a wine. When grape juice does not have a sufficient potential alcohol content (the amount of alcohol that will be potentially produced if fermented to dryness) to reach the ethanol concentration of at least 8.5% *v*/*v* required for wine stability [1], it is necessary to increase its sugar concentration by adding dry sugar (sucrose), CM, or RCM [2].

CM and RCM are concentrated solutions of whole grape sugar, used for the enrichment of grape must, grape must in fermentation, and new wine still in fermentation. RCM can also be used to sweeten wines and for fermentation in the production of sparkling wines.

CM is obtained via the partial dehydration of grape must. Its main components are glucose and fructose, but non-negligible amounts of organic acids (tartaric, malic, and citric), phenolic compounds, metals, and vitamins are also present [3]. RCM is a colorless liquid produced through the elimination of cations, anions, and phenolic compounds from the must by means of ion-exchange resins and subsequent concentration under vacuum. The resulting syrup is a mixture of glucose, fructose, and trace substances, with a soluble solid content of approximately 68–70 °Brix [4].

The low residual impurities make RCM the ideal enrichment product because its use respects the varietal identity of grape musts without altering the sensory characteristics of wine. While the addition of CM and RCM is always permitted in all European countries, the use of sucrose is only legal for specific wine-growing regions and vintages in different European (e.g., France, Germany, Austria, Poland, England, and Hungary) and extra-European (e.g., Australia) countries [5]. In Italy, as in Spain, Portugal, and Greece, the addition of exogenous sugar by unscrupulous wine producers is forbidden and constitutes food fraud [6].

The high costs of the production process and the grape raw material encourage adulteration in RCM with lower-cost inverted sucrose obtained from plants other than grapes, especially beet or cane. Since 1990, the International Organisation of Vine and Wine (OIV) has issued specific official isotopic methods (OIV MA-AS-311-05 and MA-AS-312-06) to fight against this practice [7]. They are based on the analysis of the stable isotopic ratios of hydrogen (D/H) and carbon (^13^C/^12^C, expressed as *δ*^13^C) in ethanol distilled after fermentation. These two parameters are completely different depending on whether the ethanol comes from grapes or from exogenous sugars such as beets and cane, due to the different photosynthetic cycles (C3 or C4) of the plants [8].

As reported by Christoph et al., the use of this technique has limitations, especially in the identification of mixtures of grape sugars with medium or low concentrations of adulterating sugars (e.g., sucrose or sugar syrups from beet or cane), and therefore it is necessary to expand the analytical approaches in order to guarantee the authenticity of CM and RCM [9].

*Myo*- and *scyllo*-inositol are two minor sugars characteristic of grape must [10,11,12], which are not retained by the resins used for the concentration process used to obtain CM and RCM, and these two polyalcohols are not naturally present in other commercial sugar syrups from different botanical origins (e.g., sucrose from beet, cane or sugar syrup from fruits, etc.). Furthermore, *scyllo*- and *myo*-inositol have been described as stable in terms of the storage or processing of juice [13]. As reported by Monetti et al., the content of these tracers and the measure of their ratio integrate the information provided by official OIV methods and could contribute to the characterization of the genuineness of CM and RCM and to achieving an objective classification in the most difficult cases of adulteration [14].

In the definition of “rectified concentrated must”, Regulation (EU) No. 1308/2013 adopted the proposed parameters only partially, focusing only on the necessary presence of *myo*-inositol, without considering *scyllo*-inositol and the ratio between the two isomers [15].

For Italian products, a minimum content of *myo*-inositol and *scyllo*-inositol below which a dilution of CM and RCM with non-adulterating grape sugars free from *myo*-inositol and *scyllo*-inositol can be identified has never been clearly defined. In the literature, there is only one article by Versini et al., published in the 1980s in an Italian journal, which concerns a small number of RCM samples. In that study, a minimum content of 750 mg/kg of sugar for *myo*- and 30 mg/kg of sugar for *scyllo*-inositol and a concentration ratio between *myo*- and *scyllo*-inositol equal or lower to 20 were reported [16].

For this reason, in this study, for the first time, we investigated the concentration variability of *myo*- and *scyllo*-inositol directly in natural Italian grape musts of different varieties and vintages. Furthermore, the possible impact of the concentration process used to produce CM on the native content of *myo*-inositol and *scyllo*-inositol was investigated, in order to evaluate whether the proposed concentration limits deduced from the analysis of genuine musts were reliable markers of CM and RCM authenticity.

An extensive data bank was created by analyzing 450 authentic grape musts from 17 different Italian regions. In order to describe the natural variability, the sampling was carried out during the harvest season in 2019, 2020, and 2021, and a total of 85 different grape varieties were considered.

Besides *myo*- and *scyllo*-inositol, other mono- and disaccharides were also quantified in grape must samples, such as sorbitol, sucrose, lactose, maltose, and isomaltose. The presence of these minor carbohydrates in CM and RCM can be an additional parameter of authenticity but is not regulated at the EU level, except for sucrose, whose concentration must be below the LOD set by the analytical method used [15].

All the collected samples were analyzed in terms of their content in *myo*- and *scyllo*-inositol and other minor carbohydrates in a collaborative study between two labs, to evaluate the effect of different analytical approaches. Inter-laboratory comparison measurements were conducted following two different derivatization methods and using two different gas chromatographic detectors: a mass spectrometer (MS) and a flame ionization detector (FID). The results of the two laboratories were compared to verify the reproducibility and reliability of the method.

## 2. Results and Discussion

### 2.1. Inter-Laboratory Comparison and Method Validation

**Figure 1:**
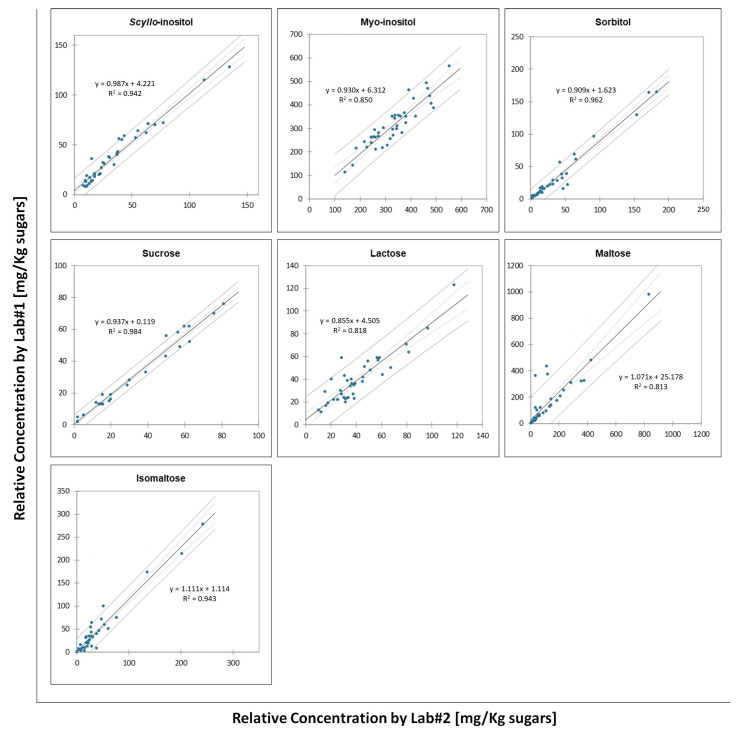
Comparison of the relative concentrations of target compounds analyzed in the 40 comparison samples at each of the two laboratories. The solid grey lines are the confidence limits for the prediction (95%), while the dotted grey lines are the confidence limits for the regression line (95%).

The collected samples were analyzed in a collaborative study involving two laboratories: Fondazione Edmund Mach (Lab#1) and Unione Italiana Vini (Lab#2). For the inter-laboratory study, 40 samples were independently analyzed by both laboratories, and the inter-comparison data are reported in Figure 1.

Correlations of results were expressed by linear regression plots for each target compound, and the slopes and square coefficient correlations (R^2^) were calculated to evaluate the agreement between the two laboratories. Lab#1 results show small negative deviations, with slopes slightly below 1 for *scyllo*-inositol, *myo*-inositol, sorbitol, sucrose, and lactose. By contrast, small positive deviations were observed for maltose and isomaltose. The R^2^ for each compound was above 0.8 (Figure 1), which demonstrated that the results of the two laboratories were highly comparable. *Scyllo*-inositol, sorbitol, sucrose, maltose, and isomaltose showed the best agreement, with R^2^ values higher than 0.9 and average relative standard deviations (RSDs) between 0.5% and 7.7%. By contrast, the R^2^ values of *myo*-inositol and lactose were slightly lower than those of the other compounds (not higher than 0.85) with RSDs of 4.9% and 1.4%, respectively. The quantitative deviations between the two laboratories are in agreement with the RSD values obtained through an inter-laboratory comparison reported in the official method for *scyllo*-inositol, *myo*-inositol, and sucrose [17].

**Figure 2:**
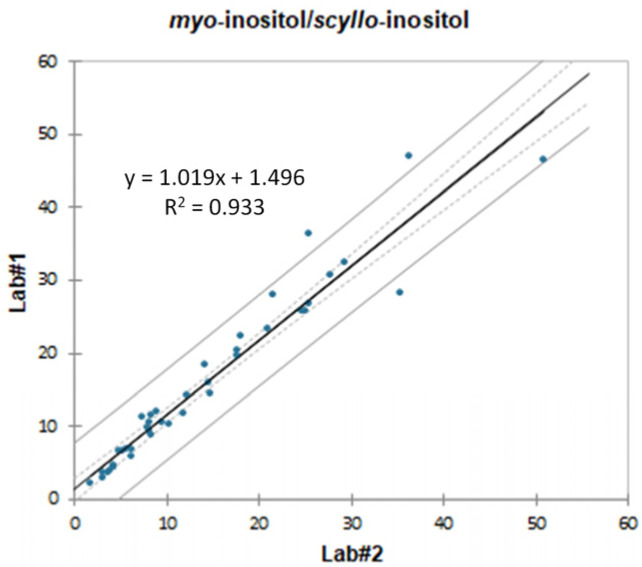
Comparison of the *myo*-inositol/*scyllo*-inositol ratio calculated for the 40 comparison samples by each of the two laboratories. The solid grey lines are the confidence limits for the prediction (95%), while the dotted grey lines are the confidence limits for the regression line (95%).

The low quantitative deviation between the laboratories for the two polyols reflects positively on the *myo*-inositol/*scyllo*-inositol parameter. The linear regression plot in Figure 2 shows a good correlation between Lab#1 and Lab#2, with an R^2^ higher than 0.9 and an average RSD of 15%.

The method was validated on the basis of Regulation (EC) 657/2002 [18] concerning the performance of analytical methods and the interpretation of results, by defining linearity (R^2^), the limit of detection (LOD), the limit of quantification (LOQ), recovery, repeatability, and reproducibility.

The signal response was evaluated at six concentration levels across a range of 5 to 650 mg/L. Analysis was performed in triplicate for every concentration level, and calibration curves were developed for each of the considered compounds. Calibration plots were drafted by plotting the relative analyte-to-IS peak area ratio against the relative analyte-to-IS concentration ratio, and the linearity was evaluated using the squared correlation coefficient (R^2^). The validation parameters (R^2^, slope, LOD, LOQ, and recovery at two concentration levels) obtained at the two laboratories are reported in Table 1.

The R^2^ values obtained for each compound were always equal to or higher than 0.995 for both laboratories; this indicated a good fit and linearity for the calibration curves in relation to the scope of the method. The slope of the regression equations was different between Lab#1 and Lab#2, due to the different detectors used. The LOQ and LOD were estimated according to the “Harmonized guidelines for single-laboratory validation of methods of analysis” (IUPAC Technical Report) [19]. Specifically, the LOD was calculated as 3 × S_0_, where S_0_ was the precision estimate of six independent determinations of analyte concentration at the lowest calibration point, while the LOQ was computed as 3×LOD. LOD and LOQ were expressed in mg/L, and they were obtained from the lowest calibration point after derivatization.

The LOQ was found to be in the range of 1.0 to 2.0 mg/L for Lab#1 and 0.5 mg/L for Lab#2. The lowest LOQ values obtained in Lab#2 are justified by the higher sensitivity of the MS detector compared with FID.

In order to determine the recovery rate, a previously analyzed grape must sample (89 mg/kg sugar of *scyllo*-inositol, 814 mg/kg sugar of *myo*-inositol, 12 mg/kg sugar of sorbitol, 12 mg/kg sugar of sucrose, 153 mg/kg sugar of lactose, 89 mg/kg sugar of maltose, and 20 mg/kg sugar of isomaltose) was spiked with a stock solution containing the target compounds at two different levels (50 and 300 mg/kg of sugar for each target compound). The spiked samples were analyzed with every analytical sequence, for a total of 15 repetitions, and the average recovery percentages are reported in Table 1. The accuracy, expressed as recovery percentage, was considered acceptable when falling within ±20% of the theoretical concentration [18]. The recovery values were between 95% and 112% for both laboratories.

The obtained results show that the two analytical approaches can be considered alternative methods. A revision of the official method (Resolution OIV-OENO 419C-2015) that also includes the method based on the use of an MS detector is therefore desirable.

To estimate the repeatability of the method for the quantification of *myo*- and *scyllo*-inositol and sucrose, two RCM samples (RCM 1 and RCM 2) with different concentrations of *scyllo*-inositol, *myo*-inositol, and sucrose were consecutively analyzed 10 times. Specifically, the concentration of *scyllo*-inositol was 298 ± 32 mg/kg of sugar in RCM 1 and 83 ± 5 mg/kg of sugar in RCM 2, and that of *myo*-inositol was 1540 ± 94 mg/kg of sugar in RCM 1 and 105 ± 7 mg/kg of sugar in RCM 2, whereas the concentration of sucrose was 12 ± 0.8 mg/kg of sugar in RCM 1 and 1 ± 0.1 mg/kg of sugar in RCM 2. The calculated repeatability was 8 mg/kg of sugar for *scyllo*-inositol, 92 mg/kg of sugar for *myo*-inositol, and 0.27 for sucrose in RCM 1, whereas it was 4 mg/kg of sugar for *scyllo*-inositol, 97 mg/kg of sugar for *myo*-inositol, and 0.29 for sucrose in RCM 2. These values were lower than those reported in the official OIV method.

To estimate the reproducibility followed by uncertainty of the method, the same RCM 1 and RCM 2 samples were analyzed 32 times (weekly for eight months).

The analytical uncertainty was calculated as the 2σ relative standard deviation of reproducibility. To allow the comparison between the estimated uncertainty and that reported in the official method, both were calculated or recalculated with respect to the fixed contents of *myo*-inositol, *scyllo*-inositol and sucrose equal to 750 mg/kg, 37 mg/kg, and 2 mg/kg, respectively. The obtained values (RCM 1: 8 mg/kg of sugar for *scyllo*-inositol and 92 mg/kg of sugar for *myo*-inositol; RCM 2: 4 mg/kg of sugar for *scyllo*-inositol and 97 mg/kg of sugar for *myo*-inositol) were lower than those calculated on the basis of the values reported in the official method (Resolution OIV-OENO 419C-2015), respectively, 14 mg/kg of sugar for *scyllo*-inositol and 106 mg/kg for *myo*-inositol. The analytical uncertainty of the official method was chosen as the uncertainty value in the subsequent evaluation of the limits (see Section 2.3 and Section 2.5 for the natural Italian grape must and for the three Italian growing regions, respectively). Its value was recalculated each time on the basis of the concentration of the analyte.

### 2.2. Influence of Concentration on the Polyalcohol Content

CMs were derived from the natural grape must through a concentration process preceded by a purification step with ion-exchange resins in the case of RCMs, as described in Section 1. Monetti et al. reported that *myo*- and *scyllo*-inositol are not lost during the CM and RCM production process, and therefore they can be used as genuineness parameters [14]. In this work, the conservation of *myo*- and *scyllo*-inositol was verified in order to confirm the possibility to apply the authenticity parameters defined in this study for grape musts to CMs and RCMs as well.

Three natural grape musts of different varieties (GM 1, GM 2, and GM 3) were collected and concentrated in triplicate to a °Brix value of 60–70 to obtain the corresponding CMs. The adopted method simulated the industrial process on a laboratory scale [20] and consisted of obtaining the concentrations using a rotary evaporator (R-205, Buchi, Milan, Italy) at 80 °C and reduced pressure (40 mbar).

The content in *myo*- and *scyllo*-inositol was determined in triplicate in Lab#1 before and after the concentration, and the results are reported in Table 2. The results indicate that the content in the two polyols as well as the ratio between their concentration are not affected by the CM production process, as demonstrated by the non-significant differences (*p* > 0.05) between the natural grape must and CM after concentration.

### 2.3. Content of Polyols and Minor Carbohydrates in Grape Musts

The concentrations of *scyllo*-inositol, *myo*-inositol, sorbitol, sucrose, lactose, maltose, and isomaltose in the 450 collected grape must samples were determined, which are summarized in Table 3. As explained in Section 3.3 the concentration values were normalized to the sugar content by expressing them as mg/kg of total sugars (mg/kg sugar).

*Myo*-inositol was confirmed as the most present minor sugar in grape must, with average values of 1472 mg/kg of sugar. All the other sugars had average values always below 161 mg/kg of sugar, with the exception of maltose, with a value of about 232 mg/kg of sugar. In a previous study on Italian musts, Perini et al. reported lower average contents of maltose (170 mg/kg of sugar), while the sucrose content between the two studies is not dissimilar (100 mg/kg of sugar vs. 65 mg/kg of sugar here reported) [21].

The concentration ranges of the target compounds are presented in Table 3. Considering the analytical uncertainty reported in the official method (see Section 2.1), which was recalculated on the basis of the concentration of the analyte, the quantities of both polyalcohols measured in the samples in this study were found to be equal to or above the limit reported for RCM by Versini et al. [16].

**Figure 3:**
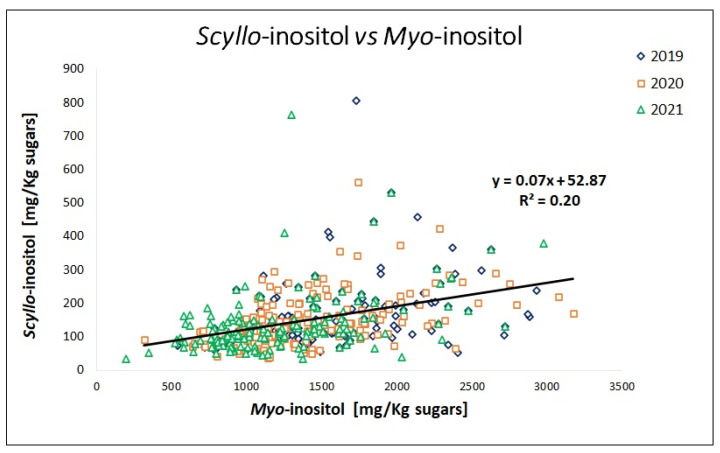
Linear correlation graph of *scyllo*-inositol with *myo*-inositol in grape musts. Blue (**◊**), red (**□**), and green (**Δ**) points correspond to the harvest seasons in 2019 (*n* = 140), 2020 (*n* = 160), and 2021 (*n* = 150), respectively.

In Figure 3, the correlation between the two parameters (*scyllo*-inositol and *myo*-inositol) was assessed. Nowadays, it is known that *myo*-inositol in plants directly derives from D-glucose-6-phosphate [22]. The conversion involves the cyclization of D-glucose-6-phosphate to inositol-3-phosphate, the loss of phosphate, and the final release of free *myo*-inositol [23]. Although *scyllo*-inositol is derived from the epimerization of *myo*-inositol through a series of enzymatic reactions [24], no linear correlation was found between their concentration in grape must samples (Figure 3). It is therefore possible to hypothesize that the activation of the biosynthetic pathway that transforms *myo*-inositol into *scyllo*-inositol is activated only under specific conditions.

### 2.4. Seasonal and Geographical Effects on the Variability of Polyols and Minor Carbohydrates

**Figure 4:**
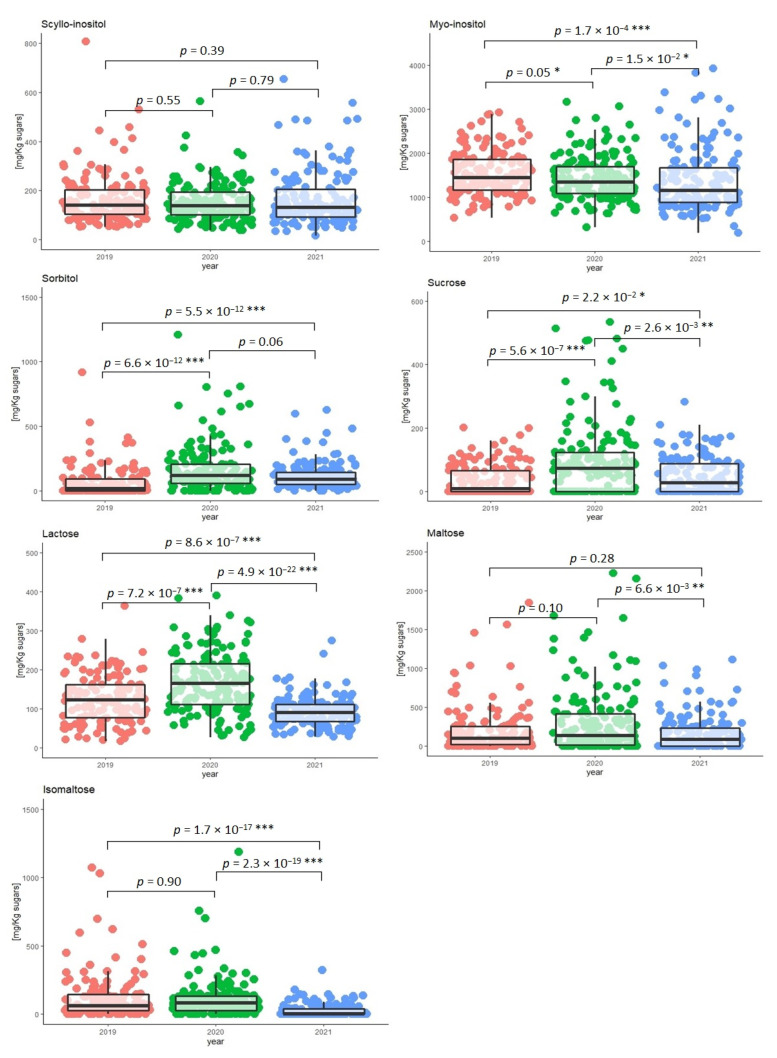
Box plot of the concentrations of *scyllo*-inositol, *myo*-inositol, sorbitol, sucrose, lactose, maltose, and isomaltose measured in the grape must samples collected in the harvest seasons in 2019, 2020, and 2021 (• 2019, *n* = 140; • 2020, *n* = 160; • 2021, *n* = 150). Significance level: * *p* ≤ 0.05 significant; ** *p* ≤ 0.01 very significant; *** *p* ≤ 0.001 highly significant.

The seasonal effect on the synthesis and accumulation of *scyllo*-inositol and *myo*-inositol and the other minor carbohydrates quantified in the grape must samples was investigated. Figure 4 shows the concentration distributions of *scyllo*-inositol, *myo*-inositol, sorbitol, sucrose, lactose, maltose, and isomaltose in the grape must samples based on the harvest year.

The post hoc Tukey HSD test was carried out to compare the concentration values, setting the significance level at 0.05. The results of the statistical test indicated significant differences (*p* < 0.05) between the three years for *myo*-inositol, sucrose, and lactose, while no statistical differences (*p* > 0.05) were found for *scyllo*-inositol. For sorbitol, no significant differences were found between 2000 and 2021, nor for isomaltose between 2019 and 2020, or for maltose between 2019 and 2020 and between 2019 and 2021.

**Figure 5:**
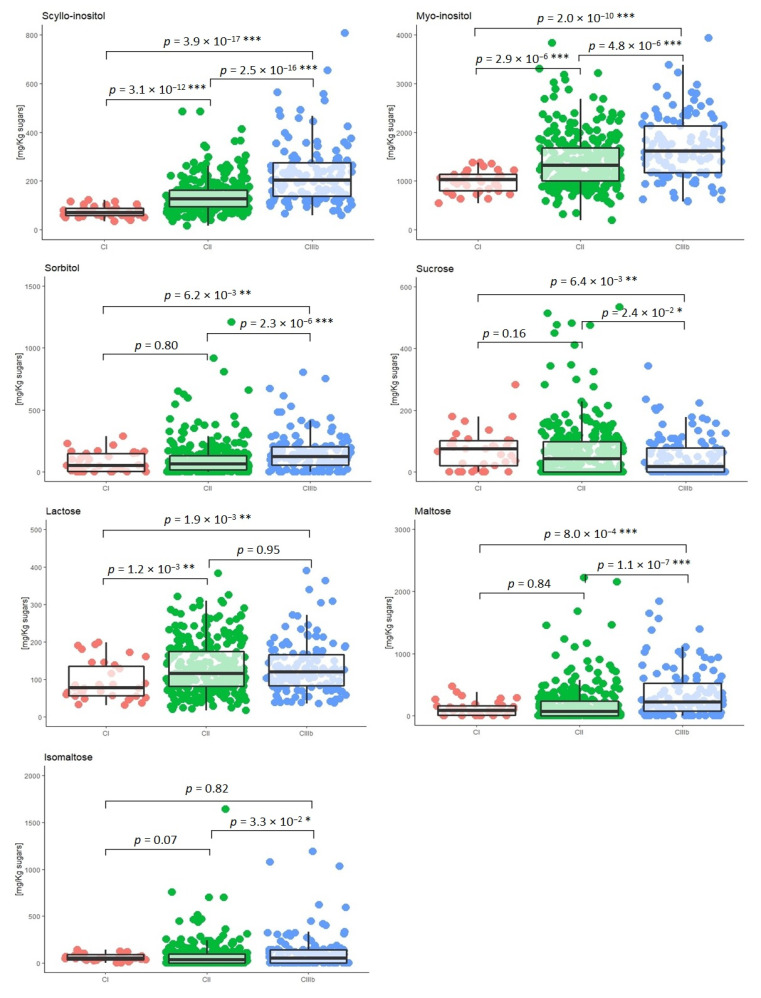
Box plot of the concentration of *scyllo*-inositol, *myo*-inositol, sorbitol, sucrose, lactose, maltose, and isomaltose measured in the three Italian wine-growing regions (• CI: *n* = 34, • CII: *n* = 290, and • CIIIb: *n* = 126). Significance level: * *p* ≤ 0.05 significant; ** *p* ≤ 0.01 very significant; *** *p* ≤ 0.001 highly significant.

The impact of the geographical origin on the content of *scyllo*-inositol, *myo*-inositol, sorbitol, sucrose, lactose, maltose, and isomaltose was studied considering three Italian wine-growing regions: CI (Trentino-Alto Adige); CII (Abruzzo, Campania, Emilia Romagna, Friuli Venezia Giulia, Lazio, Lombardy, Marche, Piedmont, Tuscany, Umbria, and Veneto); and CIIIb (Basilicata, Calabria, Apulia, Sardinia, and Sicily). The concentration distribution of the seven minor sugars was plotted by dividing the grape must samples according to the wine-growing region of provenance (Figure 5).

Statistically significant differences were found for *scyllo*-inositol and *myo*-inositol (*p* < 0.001) between the three growing regions. For sorbitol, sucrose, and maltose, significant differences were found between CI and CIIIb zones and between CII and CIIIb zones. For lactose, significant differences were found between CI and CII and between CI and CIIIb, while for isomaltose, these were only observed between CII and CIIIb zones. As shown in Figure 5, the concentration of minor sugars increased from the CI zone to the CIIIb zone. This is probably due to the different climatic conditions in the three areas considered, with higher mean temperatures during the summer before harvest in southern Italy than in northern regions [25].

Bock et al. [26] monitored the relationships between yields, grape sugar content, and temperature over two centuries (1805–2010) and, based on long-term trends, found that temperature increases have an effect on both the harvest volume and sugar content in grapes. The correlation is such that the Huglin Index, determined in France, and used for the quantification of the weather impact on the sugar content of grapes, is precisely based on the average daily temperature, the maximum daily temperature, and the latitude of the site.

### 2.5. Identification of Lower Limits for Myo- and Scyllo-Inositol and Higher Limits for Other Minor Sugars

Considering the impact of geographical origin on the content of *myo*- and *scyllo*-inositol (see Section 2.4), the lower and higher limits (the minimum and maximum values of the entire dataset) for these parameters and the mean ratios of concentration are reported in Table 4 on the basis of Italian wine-growing regions CI, CII, and CIIIb.

As shown in Table 4, regardless of geographical origin, *scyllo*-inositol concentrations are not lower than the limit reported by Versini et al. in any of the samples [16]. The samples from the CIIIb wine-growing area were found to have significantly higher values of *scyllo*-inositol that never dropped below 60 mg/kg of sugar.

The contents of *myo*-inositol varied according to the wine-growing area. The samples from the CI zone (all from the Trentino Alto Adige region) had a lower limit of 645 mg/kg of sugar. This content is lower than the limit reported by Versini et al. of 750 mg/kg of sugar [16]. On the other hand, samples from the CII and CIIIb zones met this limit, with values not lower than 756 mg/kg of sugar for musts from the CIIIb wine-growing zone.

The production of CM and RCM is mainly based on the transformation of the surplus of wine production from southern Italy and Spain, also with a view to greater price control [20]. Furthermore, considering the fact that the high cost per quintal of grapes from Trentino (and northern Italy in general) makes the transformation of these grapes into CM or RCM not economically sound [27], it is possible to identify a value of not less than 34 mg/kg of sugar for *scyllo*- and 756 mg/kg of sugar for *myo*-inositol as the lower limits for authentic CM and RCM musts.

The ratio between the concentrations of *myo*- and *scyllo*-inositol decreased from 15 to 9, from the CI zone to the CIIIb zone. The mean *myo*-inositol/*scyllo*-inositol value calculated for the entire data bank was 11, with an upper limit of 30.

Table 3 shows the maximum limits of the content of various minor sugars in RCM musts. Fraudulent practices see the increasingly massive use of sugar syrups of various origins [28]. Among the most used are those from fruit (e.g., dates), which have an isotopic composition similar to that of grapes and are therefore difficult to identify through the analysis of stable isotopes [29]. Normally, these sugar syrups provide evidence of the absence of *myo*-inositol or *scyllo*-inositol or both, as well as the presence of other minor sugars in high or very high concentrations.

The maximum limits given here can be a useful tool for identifying the illegal partial or total replacement of grape must with sugar syrup with these minor sugars as constituents or contaminants. For example, glucose/fructose syrup from maize maltose is a possible adulterant. Maltose is hydrolyzed to glucose and then converted into fructose through enzymatic means. Maltose remains an impurity, with an average percentage of 20% and a minimum of 3% to 4%. For this reason, Perini et al. suggested that the content of maltose in grape musts higher than the natural limit could be useful to identify the fraudulent addition of this syrup [21].

## 3. Materials and Methods

### 3.1. Chemicals and Reagents

For this study, *yo*-inositol (≥99%), *scyllo*-inositol (≥98%), D-(-)-sorbitol (≥99%), D-(+)-sucrose (≥99.5%), D-(+)-lactose (≥99.5%), D-(+)-maltose (≥99%), isomaltose (≥98%) xylitol (≥99%), and ethanol (≥99.8%) were purchased from Merck (Darmstadt, Germany). The silylating reagents including a mixture of hexamethyldisilazane (HMDS)/chlorotrimethylsilane (TMCS)/pyridine (2:1:10, *v*/*v*/*v*) and a mixture of N,O-Bis(trimethylsilyl)trifluoroacetamide (BSTFA) with 10% (TMCS) trimethylchlorosilane and pyridine (≥99.8%) were obtained from Sigma-Aldrich (Milan, Italy).

### 3.2. Sampling

The 450 grape must samples of 85 different varieties were collected during the harvest season in 2019, 2020, and 2021 from 17 Italian regions (Abruzzo, Basilicata, Calabria, Campania, Emilia Romagna, Friuli Venezia Giulia, Lazio, Lombardy, Marche, Piedmont, Apulia, Sardinia, Sicily, Tuscany, Trentino-Alto Adige, Umbria, and Veneto).

Sampling was carried out by collecting at least 2 kg of grapes of each variety, representative of the entire parcel, at the technological ripeness. The compositional variability within each parcel was determined by sampling one or two bunches from vines placed in each of the four corners and at the center of the plot. The bunches, collected in plastic bags, were squeezed manually, and the free-run musts thus obtained were frozen (−20 °C) and stored until the analysis.

The samples were divided into two groups, and each of the two subsets (225 samples) was separately subjected to analysis in 2 independent laboratories: Fondazione Edmund Mach, San Michele all’Adige, Trento, Italy (Lab#1), and Unione Italiana Vini, Verona, Italy (Lab#2). For the interlaboratory comparison, 40 samples from both laboratories were tested.

### 3.3. Sugar Derivatization and GC Analysis

The quantification of *myo*-inositol, *scyllo*-inositol, sorbitol, sucrose, lactose, maltose, and isomaltose was performed via gas chromatography after silylation. Both laboratories followed similar procedures based on the official method concerning the quantification of *myo*- and *scyllo*-inositol in RCM [17], with some modifications. Lab#1 used the FID, as reported in the official method, whereas Lab#2 used an MS as the detector.

To allow the comparability of the data not only for the control of musts but also of CM and RCM, the concentration values were normalized to the sugar content by expressing them as mg/kg of total sugar (mg/kg sugar). For this reason, the content of total sugar was quantified in g/kg of grape must using a WineScan ™ FT 120 spectrophotometer (Foss, Hillerød, Denmark).

An RCM sample was analyzed on a weekly basis for eight months in order to estimate the analytical uncertainty of *myo*- and *scyllo*-inositol and for sucrose extraction and analysis, based on the extended standard deviation of reproducibility.

#### 3.3.1. Lab#1 Analytical Method

An aliquot of 40 µL of each grape must sample was placed in a GC vial, and 20 µL of an internal standard solution (xylitol at 500 mg/L in water) was added. The sample was dried under a gentle stream of nitrogen at room temperature, adding 100 µL of ethanol to facilitate evaporation. The derivatization of polyols and sugars was effected with 400 µL of the silylation mixture HMDS/TMCS/pyridine (2:1:10, *v*/*v*/*v*), for 1 h at 70 °C.

The silylated samples were analyzed with a Clarus 500 gas chromatograph (PerkinElmer^®^, Inc., Waltham, MA, USA) connected to an FID, injecting 1 µL in split mode (5:1) into a DB-5MS (30 m × 0.25 mm × 0.25 μm) capillary column. The injector was set at 300 °C and the detector at 250 °C. The oven temperature was programmed starting at 100 °C for 1 min, raised to 200 °C by 10 °C/min increments, held for 1 min, and finally raised to 280 °C by 2.5 °C/min increments and held at this temperature for 5 min. The flow rate of the carrier gas (hydrogen) was maintained at 1 mL/min. The identification of compounds was achieved by comparing their retention times with those of authentic compounds.

Calibration levels were prepared by spiking a grape must sample with different volumes of the stock solution containing *myo*-inositol (1016 mg/L), *scyllo*-inositol (182 mg/L), D-(-)-sorbitol (334 mg/L), D-(+)-sucrose (296 mg/L), D-(+)-lactose (622 mg/L), D-(+)-maltose (426 mg/L), and isomaltose (160 mg/L). The final concentration was between 2 and 70 mg/L for *scyllo*-inositol; 5 and 180 mg/L for D-(-)-sorbitol, D-(+)-sucrose, and isomaltose; 10 and 300 mg/L for D-(+)-maltose and D-(+)-lactose; and between 20 and 650 mg/L for *myo*-inositol.

#### 3.3.2. Lab#2 Analytical Method

Briefly, 40 µL of grape must and 20 µL of the internal standard solution (xylitol at 500 mg/L in water) were dried in a vial under a gentle stream of nitrogen and left at 40 °C overnight. The residue was dissolved in 120 µL of pyridine and silylated with 120 µL of BSTFA + 10% TMCS for 70 min at 70 °C. The GC analysis was performed by injecting 2 µL in split mode (15:1) in an Agilent 6890 gas chromatograph coupled with an Agilent 5975 mass spectrometer and equipped with a DB-5MS (30 m × 0.25 mm × 0.25 μm) capillary column. The carrier gas (He) was set at 1.5 mL/min.

The injector was set at 250 °C and the source at 230 °C. The oven temperature was programmed starting at 150 °C for 40 min, raised to 280 °C by 5 °C/min increments, and finally raised to 300 °C by 40 °C/min increments and held at this temperature for 10 min.

The mass spectrometer operated in the electron ionization mode (positive ion, 70 eV), and the mass spectra were acquired in the full-scan mode from 50 to 500 *m*/*z*. For the identification and quantification of the target analytes, the selected ion-monitoring chromatograms were obtained from the acquired signal by selecting the most abundant characteristic fragment. The characteristic fragments were 217 *m*/*z* for xylitol; 319 *m*/*z* for sorbitol; 318 *m*/*z* for *scyllo*-inositol; 305 *m*/*z* for *myo*-inositol; 361 *m*/*z* for sucrose; and 204 *m*/*z* for lactose, maltose, and isomaltose. The calibration levels were prepared similarly to those of Lab#1.

### 3.4. Statistical Analysis

Data analysis and graphical representation were performed using R (R Foundation for Statistical Computing, Vienna, Austria) version 4.0.3 in RStudio (RStudio 2021.09.0 Inc., Boston, MA, USA).

The post hoc Tukey HSD test was carried out to compare the concentration values between the three harvest seasons and the three growing regions.

## 4. Conclusions

In this study, we identified, for the first time, the possible limits of the authenticity of the content of *myo*- and *scyllo*-inositol and various minor sugars. A *myo*- and *scyllo*-inositol content below the reported limits could indicate a dilution or replacement of the grape must with other sugary products naturally free of these two polyalcohols.

Minor sugar concentrations higher than the natural ones identified in this study could indicate the possible addition of sugar syrups containing these minor sugars as components to the grape must. The demonstrated stability of the polyalcohols during the CM and RCM concentration phase enables the use of the limits identified for the Italian must for the control of concentrated products as well.

The concentrations of *myo*- and *scyllo*-inositol were not correlated to each other and varied according to the geographic origin of the product, with tendentially higher contents in the southern Italian regions, probably due to having different climatic conditions.

As demonstrated, the applied analytical method (GC-FID vs. GC-MS) had no effect on the results, and a revision of the official OIV method that includes both analytical approaches is therefore desirable.

## Figures and Tables

**Table 1:** Validation parameters: correlation coefficient (R^2^), slope, LOD, LOQ, and recovery.

Compound	Correlation Coefficient (R^2^)		Slope ± STD		LOD (mg/L Grape Must)		LOQ (mg/L Grape Must)		Recovery (%) Low Level		Recovery (%) High Level	
	Lab#1	Lab#2	Lab#1	Lab#2	Lab#1	Lab#2	Lab#1	Lab#2	Lab#1	Lab#2	Lab#1	Lab#2
sorbitol	0.997	0.998	0.0039 ± 0.0005	2.04 ± 0.20	0.7	0.2	2.0	0.5	95	104	97	104
*scyllo*-inositol	0.998	0.998	0.0058 ± 0.0004	4.07 ± 0.25	0.3	0.2	1.0	0.5	99	112	105	112
*myo*-inositol	0.999	0.997	0.0053 ± 0.0003	3.92 ± 0.44	0.3	0.2	1.0	0.5	105	100	96	100
sucrose	0.997	0.995	0.0014 ± 0.0009	2.10 ± 0.81	0.7	0.2	2.0	0.5	102	99	104	99
lactose	0.996	0.997	0.0025 ± 0.0011	2.85 ± 0.73	0.7	0.2	2.0	0.5	97	99	101	99
maltose	0.998	0.996	0.0022 ± 0.0008	3.56 ± 0.83	0.7	0.2	2.0	0.5	95	98	95	98
isomaltose	0.997	0.997	0.0022 ± 0.0009	3.07 ± 0.87	0.7	0.2	2.0	0.5	106	100	98	100

**Table 2:** Means of *myo*- and *scyllo*-inositol content in natural grape must and corresponding concentrate (CM); values in brackets refer to standard deviations.

	Natural Grape Must				CM			
	Brix	*Scyllo*-Inositol	*Myo*-Inositol	*Myo*/*Scyllo*	Brix	*Scyllo*-Inositol	*Myo*-Inositol	*Myo*/*Scyllo*
		(mg/kg Sugar) (SD)	(mg/kg Sugar) (SD)			(mg/kg Sugar) (SD)	(mg/kg Sugar) (SD)	
**GM 1**	6.9	129 (11)	1364 (50)	11	69.2	112 (5)	1302 (15)	12
**GM 2**	5.9	104 (8)	1829 (70)	18	70.1	115 (3)	1929 (80)	17
**GM 3**	5.3	106 (7)	1322 (30)	12	67.7	111 (8)	1309 (69)	12

**Table 3:** Concentration range (mean value, max value, and min value), median, standard deviation (SD), first quartile (Q1), and third quartile (Q3) of the target compounds detected in natural grape must samples (*n* = 450).

	Concentration (mg/kg Sugar)			Median	SD	Q1	Q2
	Mean	Min	Max				
*Scyllo*-inositol	161	34	806	136	98	97	199
*Myo*-inositol	1472	645	3932	1346	565	1055	1764
Sorbitol	120	<0.7	1207	77	144	26	156
Sucrose	65	<0.7	534	37	82	11	94
Lactose	130	21	390	116	69	79	169
Maltose	232	<0.7	2222	107	332	22	299
Isomaltose	89	<0.7	1639	42	156	5	110

**Table 4:** Lower and higher concentrations, mean, median, standard deviation (SD), first quartile (Q1), and third quartile (Q3) of *myo*- and *scyllo*-inositol and mean values of the *myo*-inositol/*scyllo*-inositol ratio in the three Italian wine-growing regions (CI: *n* = 34, CII: *n* = 290, and CIIIb: *n* = 126).

Italian Wine-Growing Regions	*Scyllo*-Inositol						*Myo*-Inositol						*Myo*-Inositol /*Scyllo*-Inositol Mean
	(mg/kg Sugar) Min–Max	Mean	Median	SD	Q1	Q2	(mg/kg Sugar) Min–Max	Mean	Median	SD	Q1	Q2	
CI	34–122	73	71	23	57	89	645–1372	1000	1023	219	792	1160	15
CII	34–485	143	127	43	97	166	759–3830	1437	1321	218	1024	1685	12
CIIIb	60–806	230	203	108	139	278	756–3932	1700	1617	732	1177	2132	9

## Data Availability

The data presented in this study are available in the present article.
